# Release of Mast Cell Mediators from Cochlear Tissue Following Short Exposure to Compound 48/80 or Cisplatin, and Their Damage to Cochlear Structure

**DOI:** 10.3390/cells14201615

**Published:** 2025-10-17

**Authors:** Bin Zeng, Stefan Frischbutter, Sherezade Moñino-Romero, Jörg Scheffel, Frank Siebenhaar, Heidi Olze, Agnieszka J. Szczepek

**Affiliations:** 1 Department of Otorhinolaryngology, Head and Neck Surgery, Charité—Universitätsmedizin Berlin, Corporate Member of Freie Universität Berlin and Humboldt Universität zu Berlin, 10117 Berlin, Germany; zengbin_pumc@163.com (B.Z.); heidi.olze@charite.de (H.O.); 2 Institute of Allergology, Charité—Universitätsmedizin Berlin, Corporate Member of Freie Universität Berlin and Humboldt-Universität zu Berlin, 12200 Berlin, Germany; stefan.frischbutter@charite.de (S.F.); sherezade.monino-romero@charite.de (S.M.-R.); joerg.scheffel@charite.de (J.S.); frank.siebenhaar@charite.de (F.S.); 3 Fraunhofer Institute for Translational Medicine and Pharmacology ITMP, Immunology and Allergology, 12200 Berlin, Germany; 4 Faculty of Medicine and Health Sciences, University of Zielona Góra, 65-046 Zielona Góra, Poland

**Keywords:** ototoxicity, cisplatin, mast cells, mediators, cochlea, mouse

## Abstract

**Highlights:**

**What are the main findings?**

- The supernatant from degranulated BMMCs induces structural changes to coch-lear tissues.
- Cochlear tissues stimulated with Compound 48/80 or cisplatin release histamine and chymase.

**What is the implication of the main finding?**

- Mast cell mediators have a potential to remodel cochlear tissues.
- Resident cochlear mast cells can be stimulated during chemotherapy with cispla-tin to contribute to hearing loss by releasing proinflammatory mediators.

**Abstract:**

The cochlea is susceptible to damage from ototoxic agents such as cisplatin, yet the mechanisms underlying cochlear injury remain incompletely understood. Mast cells (MCs), key immune players in allergic and inflammatory responses, have recently been identified in the rodent cochlea and implicated in cisplatin-induced ototoxicity. Our study investigated the role of MC degranulation in cochlear damage and evaluated the activation capacity of cochlear-resident MCs. Bone marrow-derived MCs (BMMCs) were cultured and induced to degranulate via IgE-anti-DNP/DNP stimulation, and the supernatants were applied to cochlear explants. Cochlear explants were also treated with Compound 48/80 (CP48/80) or cisplatin to assess MC activation. Morphological changes were assessed and hair cells (HC) quantified via phalloidin staining, while ELISA measured mediator release. Supernatants from degranulated BMMC induced a dose-dependent HC loss and tissue damage. A significant chymase and tryptase release was triggered by CP48/80 from cochlear MCs, with chymase elevation detected even at low concentrations. Cochlear MCs were rapidly activated by cisplatin exposure, elevating chymase and histamine levels, and the effects were attenuated by the MC stabilizer sodium cromolyn. Notably, tryptase remained undetectable post-cisplatin treatment, suggesting tissue-specific MC responses. These findings establish MC degranulation as an early event in cisplatin-induced cochlear injury, mediated by chymase and histamine. Our study highlights MCs as potential therapeutic targets for mitigating ototoxicity and underscores the need to explore MC-driven pathways in hearing loss.

## 1. Introduction

The mammalian inner ear, a delicate sensory organ responsible for auditory transduction and balance [1], is susceptible to damage from diverse ototoxic insults, including aminoglycoside antibiotics (e.g., gentamicin or streptomycin) and chemotherapeutic agents, such as cisplatin [2]. Cisplatin can cause permanent, bilateral sensorineural hearing loss (SNHL) and tinnitus. Cisplatin-induced hearing loss (CIHL) typically originates in the high-frequency range and progressively extends to lower frequencies as treatment continues and cumulative cisplatin exposure increases [3,4,5]. The extent of hearing impairment correlates with the total cumulative dose administered [3,4,5]. Cisplatin has the potential to damage multiple cochlear structures, including outer hair cells (OHCs) and inner hair cells (IHCs), the stria vascularis, and the spiral ganglion [2,3,4,5,6,7]. Mechanisms contributing to CIHL development include the production of toxic reactive oxygen species and a decrease in the cochlea’s antioxidant defenses [2,3,4,5,6]. While oxidative stress and apoptosis are well-established contributors to cisplatin ototoxicity, emerging evidence suggests that neuroimmune interactions play a critical role in mediating cochlear injury. Among immune cells, mast cells (MCs), tissue-resident sentinels of allergic and inflammatory responses, have recently been identified as potential key players in inner ear pathologies [8]. Notably, MCs, which populate mucosal and connective tissues, were recently confirmed in the rodent cochlea, where they are predominantly localized to the modiolus and spiral ligament [9].

Epidemiological studies consistently highlight a clinical correlation between allergic diseases and inner ear disorders [10,11,12,13,14]. Our recent systematic review of 21 clinical studies revealed that patients with Ménière’s disease (MD), idiopathic sudden sensorineural hearing loss (ISSHL), and acute low-tone hearing loss (ALHL) exhibit significantly higher serum IgE levels compared to healthy controls, with allergen provocation exacerbating endolymphatic hydrops and auditory symptoms in sensitized individuals [15]. Roomiani et al. observed in 2022 that individuals diagnosed with MD have significantly higher circulating IgE concentrations than the control group, and that this is associated with upregulated immunoreactivity in serum against common inhalant and food allergens [15]. Frejo et al. confirmed this observation in 2025 by finding that 25% of MD have elevated plasma IgE levels and that their plasma polarizes the differentiation of the monocytic cell line (THP-1) toward the M2 phenotype [16]. In line with these studies, Kothandaraman et al. found that more than half of 25 patients with MD reacted to common food and respiratory allergens [17].

MCs, key mediators of allergic reactions and immune responses, are implicated in diverse pathologies ranging from pulmonary infections [18] to neural inflammation [19]. In addition to the findings on resident MCs in the rodent cochlea, our research group observed cisplatin-induced quantitative changes in cochlear explants [9], followed by the critical discovery that cisplatin triggers MC degranulation, leading to auditory HC loss—a process that can be inhibited by the MC stabilizer sodium cromolyn [7]. While these findings confirm MC involvement in cochlear dysfunction, the specific mechanisms behind MC-mediated inner ear damage remain unclear.

In our previous studies on cochlear MCs, we identified their anatomical location and the common markers they express (high-affinity immunoglobulin epsilon receptor subunit alpha (FcεRIα), chymase, tryptase, CD117, and heparin) [9]. We also showed that cisplatin causes cochlear MC degranulation, as seen under fluorescent microscopy [7]. Finally, we found that preincubation with 20 µM sodium cromolyn can reduce HCs’ damage caused by 15 µM cisplatin [7]. The current study builds on our previous work with three main goals: (1) examining the morphological effects of MC degranulation on cochlear microstructure; (2) evaluating the activation potential of cochlear MCs via the Compound 48/80 pathway; and (3) outlining the temporal patterns of cisplatin-triggered MC mediator release with pharmacological intervention. Using ex vivo explant models and mast cell-specific degranulation assays, we aim to position MC activation as an early trigger of cisplatin ototoxicity. By bridging the gap between MC-driven inflammation and cochlear damage, our results could lead to new hearing preservation strategies that combine MC-targeted treatments with traditional antioxidant therapies, thereby greatly enhancing otoprotection.

## 2. Materials and Methods

A large portion of the Section 2 was previously published in the dissertation of Dr. Zeng [20].

The experimental protocol received approval from the Governmental Ethics Commission for Animal Welfare (LaGeSo Berlin, Germany; approval numbers: T 0292/16 granted on 7 December 2016 and T-CH 0036/22 granted on 21 November 2022). The C57BL/6 mice used were procured from the local animal facility of Charité-Universitätsmedizin Berlin.

### 2.1. MC Isolation and Culture

Murine bone marrow-derived MCs (BMMCs) were obtained from C57BL/6(J) adult male and female mice aged 8–12 weeks. At first, male and female animals were tested separately (e.g., supernatant from BMMC derived from males on male cochlear explants), to address sexual dimorphism in MC biology reported in several studies [21,22]. However, our preliminary experiments have not shown differences in the outcome—likely because we used in vitro tissues from sexually immature animals for the outcome measures; therefore, to accommodate 3R guidelines, we included both male and female animals. Following euthanasia via cervical dislocation, the mouse abdomens and legs were disinfected with 70% ethanol. The skin around each leg, near the ilium, was incised and removed by pulling it toward the feet. The feet were disarticulated, and the femur and hip joints were exposed using sterile scissors and tweezers. The femur head was dislocated, and the leg was excised without fracturing bones. Extraneous tissue was removed from the femur and tibia, which were then disinfected, separated, and stored in a 50 mL tube with wash medium on ice. Bone marrow was flushed using a sterile syringe with PBS, and the collected cells were centrifuged at 300× *g* for 4–10 min at room temperature (RT). The pellet was resuspended in culture medium (1 mouse per 15–20 mL) and transferred to a tissue-treated cell culture flask, with the flask size adjusted according to the number of mice. The MC medium consisted of RPMI 1640 (#Cat.R8758-1L, Merck KGaA, Darmstadt, Germany) supplemented with 10% heat-inactivated fetal bovine serum (#Cat. FBS.S 0615 HI, Bio&Sell GmbH, Feucht, Germany), 1% penicillin/streptomycin (#Cat. 15140112, Thermo Fisher Scientific, Hennigsdorf, Germany), 1% DMEM (#Cat.11960044, Thermo Fisher Scientific), 1% sodium pyruvate (#Cat.11360070, Thermo Fisher Scientific), 10 ng/mL murine IL-3 (#Cat.213-13-500ug, Thermo Fisher Scientific), and stem cell factor (#Cat. 250-03, Thermo Fisher Scientific). Cells were incubated at 37 °C with 5% CO_2_, and the medium and flask were changed every two days until the desired density was reached.

### 2.2. MC Degranulation Assay

All reagents were pre-warmed to 37 °C. HEPES-Tyrode’s buffer (135 mM NaCl, 5 mM KCl, 1 mM MgCl_2_, 1.8 mM CaCl_2_, 5.6 mM dextrose, 20 mM HEPES; adjust to pH 7.4 with NaOH) (1 mL/well) was added to a 12-well plate. MCs were counted, diluted to 20,000 cells/well, and centrifuged at 300× *g* for 3 min at RT. Afterward, the supernatant was discarded. The pellet was gently resuspended in 500 µL of medium, and MCs were sensitized with DNP-specific IgE(α DNP clone ε26 from Dr. Fu-Tong Liu, UC Davis [23], 1 µg/mL) for 1 h at 37 °C and 5% CO_2_. After centrifugation (300× *g*, 3 min, RT) and removal of the supernatant, cells were treated with DNP (#Cat.A6661, Merck KGaA) (0.01 µg/mL) for 1 h at 37 °C, with buffer as a control. Following centrifugation, the supernatant and cell pellet were collected for further analysis.

### 2.3. Cochlear Explant Isolation

Cochlear explant cultures were prepared from postnatal days 3–5 C57BL/6 mice. Explants were obtained as previously described [7,9]. Following decapitation, the head was disinfected with 70% ethanol and positioned ventral side down. The scalp was removed, the skull was bisected along the mid-sagittal plane, and the brain was extracted to expose the posterior fossa. The temporal bones were isolated and transferred to Petri dishes containing cold, sterile DMEM/F12 (#Cat.21331-020, Thermo Fisher Scientific). Under a stereomicroscope (Carl Zeiss, Jena, Germany), the tympanic membrane and annulus were peeled away laterally, and the surrounding cartilage was removed to reveal the cochlear capsule. The capsule was either fragmented from the oval window to the apex or extracted intact from the base. The stria vascularis and spiral ligament were detached as a single unit, and the organ of Corti (OC) was separated from the modiolus.

Cochlear explants were incubated in 4-well slides (# Cat.PEZGS0416, Merck KGaA) with 500 μL of culture medium DMEM/F12 (#Cat.21041025, Thermo Fisher Scientific) supplemented with 10% fetal bovine serum (#Cat.S0113, Biochrom AG, Berlin, Germany), 1.3% glucose (#Cat.G8769, Merck KGaA), 0.2% penicillin G (#Cat. A321-42, Biochrom AG), 0.2% insulin-transferrin-selenium solution (#Cat.11207500, Roche, Basel, Switzerland), and 0.025% insulin-like growth factor (#Cat. 4326, Bio-Techne GmbH, Wiesbaden-Nordenstadt, Germany), freshly prepared and sterilely filtered (0.2 μm). Two culture methods were employed: for supernatant collection, the whole cochlear tissues were free-floating; for histological analysis, they were laid flat and secured by surface tension. Explants were incubated at 37 °C and 5% CO_2_ for 24 h before further treatment.

### 2.4. Cochlear Tissue Treatment

#### 2.4.1. Flat Cochlear Tissue Explant Treated with Supernatant from IgE-anti-DNP/DNP Stimulated BMMCS

Supernatants from the BMMC degranulation assay were collected, aliquoted, and stored at −80 °C. On the experimental day, supernatants were diluted in culture medium to a final volume of 500 µL under sterile conditions, with dilution factors detailed in the Section 3. Controls were kept in the tissue culture medium. The explants were incubated at 37 °C with 5% CO_2_ for 24 h, then fixed in 10% formalin and stored in PBS at 4 °C until the immunofluorescence was completed.

#### 2.4.2. Stimulation of Floating Cochlear Tissue with Compound 48/80

Compound 48/80 (CP48/80) (#Cat.C2313, Merck KGaA) was dissolved in distilled water to create a 50 mM stock solution. The stock solution was diluted in 500 µL of OC medium to the desired working concentrations. Free-floating cochlear explants were exposed to CP48/80 for 1 h, while control explants were kept in the medium without CP48/80. After incubation, supernatants were collected for further analysis.

#### 2.4.3. Exposure of Floating Cochlear Tissue to Cisplatin and Sodium Cromolyn

The primary stock solution of cisplatin (# Cat. 232120, Merck KGaA) was prepared by dissolving it in dimethyl sulfoxide (DMSO) to a final concentration of 100 mg/mL. Then, the stock solution of cisplatin was prepared by diluting the primary stock 1:100 in DMEM/F12 medium to 3.3 mM, and the solution was aliquoted. The 3.9 mM stock solution of sodium cromolyn (SC) (#Cat. T1260, BioCat GmbH, Heidelberg, Germany) was diluted to 20 µM. As specified in the Section 3, the cochlear explants were either exposed to 15 µM cisplatin for 1 h or pre-incubated with SC for 2 h, followed by exposure to 15 µM cisplatin. The control groups assigned to each treatment were kept in the medium throughout the entire incubation period. Supernatants were then collected for further analysis.

The concentrations of 15 µM cisplatin and 20 µM sodium cromolyn were previously determined in titration experiments and shown to effectively induce or inhibit, respectively, degranulation of cochlear mast cells [7].

### 2.5. Enzyme-Linked Immunosorbent Assay

Supernatants from cochlear cultures exposed to CP48/80, cisplatin, or control conditions were collected and analyzed for chymase (#Cat. SEG515Mu, BIOZOL Diagnostica Vertrieb GmbH, Hamburg, Germany), tryptase (#Cat.SEB070Mu, BIOZOL Diagnostica Vertrieb GmbH), and histamine (#Cat.NBP2-62860, BioCat GmbH) using ELISA. The assays were conducted according to the manufacturer’s instructions.

### 2.6. HC Quantification

HC quantification was performed on flat cochlear explants treated with supernatant from IgE-anti-DNP/DNP-stimulated BMMCs. After incubation, explants were fixed in 10% formalin (#Cat.HT5011, Merck KGaA) for 45 min at RT under a fume hood and then washed three times with wash buffer. Permeabilization was conducted using 0.5% Triton X-100 (#Cat. 9002-93-1, Merck KGaA) in PBS for 15 min. To evaluate the number and morphology of HC, explants were stained with fluorescently labeled phalloidin, which binds to filamentous actin (F-actin) and highlights stereocilia and cuticular plates through fluorescence microscopy. Phalloidin-iFluor^®^ 594 (#Cat.ab176757, Abcam, Cambridge, UK) was diluted 1:1500 in PBS, and explants were incubated at RT for 45 min, protected from light. After three washes, explants were embedded in ProLong™ Gold antifade reagent with DAPI (#Cat. 8961S, Cell Signaling Technology Europe B.V., Leiden, The Netherlands) for imaging.

The stained specimens were covered with a coverslip and left at room temperature for up to 24 h to allow the mounting solution to dry, after which they were stored at 4 °C. Imaging, quantification, and analysis were performed using an EVOS FL (Thermo Fisher Scientific) and a Keyence BZ-X800 fluorescence microscope (KEYENCE DEUTSCHLAND GmbH, Frankfurt am Main, Germany) at 40× magnification. Fluorochrome-specific filters were used to visualize emission colors. HCs were counted along 100 μm segments in five distinct regions per cochlear section. Intact HCs exhibited physiologically arranged stereocilia, whereas defective HCs showed disorganized or fused stereocilia. Cells were classified as missing if gaps were present without visible stereocilia or cuticular plates. An average HC count was determined for each explant, with at least five explants analyzed per condition.

### 2.7. Statistical Analysis

The statistical analyses were performed using the software GraphPad Prism 9 (Version 9.5.1.733, GraphPad Software, Inc., San Diego, CA, USA). The normality of data distribution was assessed using the Shapiro–Wilk test. If the data met the normality and homoscedasticity assumptions, they are presented as the mean ± standard deviation (SD), and comparisons among more than two groups were performed using one-way analysis of variance (ANOVA), post hoc comparisons were performed using Dunnett’s test for comparisons against a single control group, and Tukey’s test for all pairwise comparisons. If the data were not normally distributed, they were presented as the median and the interquartile range (IQR). The nonparametric Kruskal–Wallis test was then used for comparisons, followed by Dunn’s multiple comparisons test with adjustment for multiple testing. For each ANOVA, the F statistic and degrees of freedom are reported in the format F(df_between, df_within) = value; for Kruskal–Wallis, the H statistic is reported where applicable. A significance value (*p*-value) of less than 0.05 was considered statistically significant. n indicates the number of independent biological replicates and is stated for each figure/experiment.

## 3. Results

A large portion of the Section 3 was previously published in the dissertation of Dr. Zeng [16].

### 3.1. Cochlear Morphology Alterations Following Exposure to Supernatant from Bone Marrow-Derived MCs Stimulated with Mouse IgE-anti-DNP/DNP

**Figure 1:**
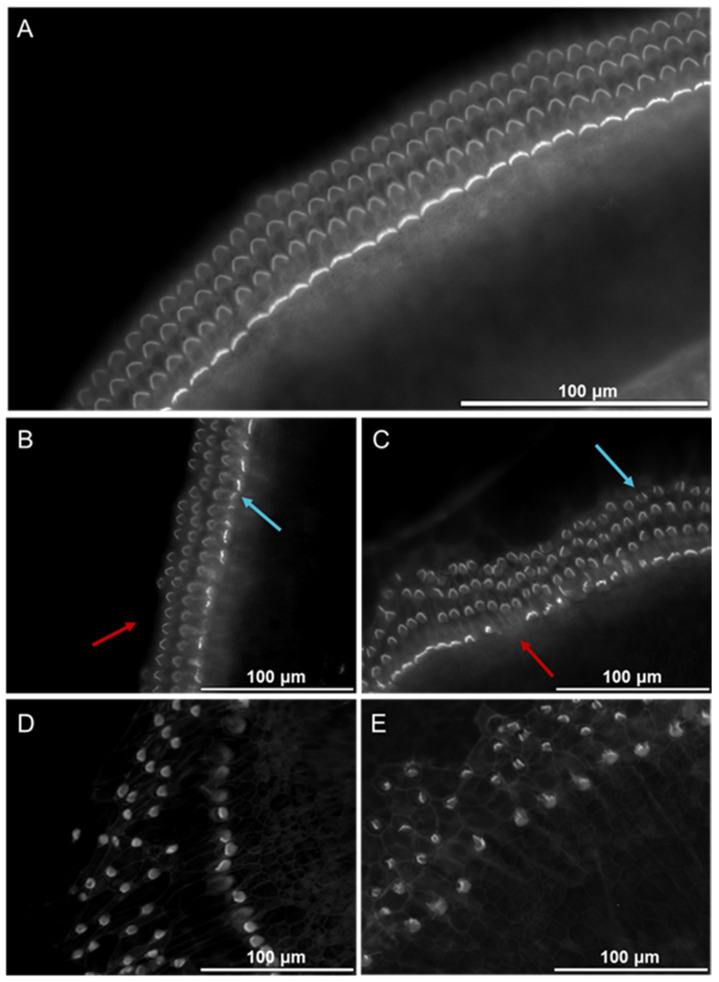
The cochlear morphology changes after exposure to the supernatant from degranulated BMMC. The micrographs show epifluorescence of phalloidin-labeled HCs in cochlear explant cultures treated with various dilutions of BMMC supernatant. Red arrowheads mark representative missing cells, and blue arrowheads mark representative damaged cells. (**A**) Control group (n = 12); (**B**) Exposure to BMMC supernatant diluted 1:2000 (n = 13); (**C**) Exposure to BMMC supernatant diluted 1:1000 (n = 7); (**D**) Exposure to BMMC supernatant diluted 1:400 (n = 11); (**E**) Exposure to BMMC supernatant diluted 1:20 (n = 10); The scale bar represents 100 µm.

**Figure 2:**
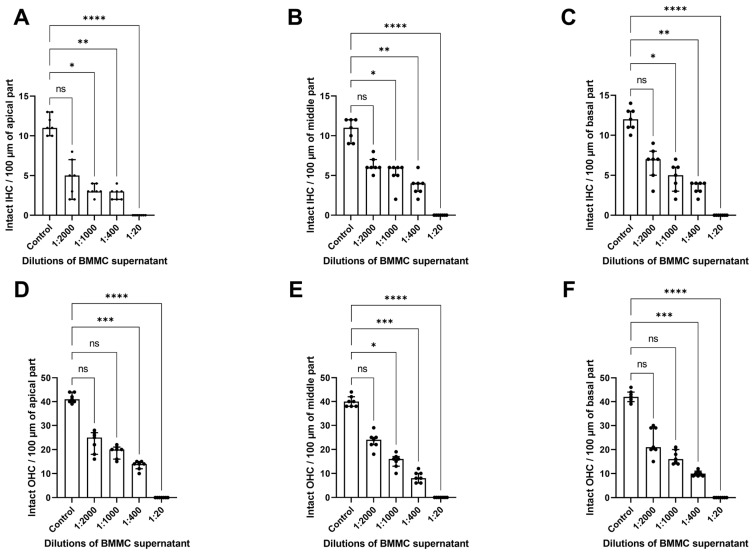
Loss of HCs due to exposure to BMMC supernatant. The number of intact IHC per 100 μm length at the apical (**A**), middle (**B**), and basal parts of the cochlea (**C**), as well as the number of intact OHC per 100 μm length at the apical (**D**), middle (**E**), and basal (**F**) parts of OC explants, were significantly reduced by incubation with MC supernatant. Data were collected from seven separate experiments and are presented as medians and interquartile ranges (IQRs). OHC, outer hair cells; IHC, inner hair cells; ns, not significant; *, *p* < 0.05; **, *p* < 0.01; ***, *p* < 0.001; ****, *p* < 0.0001 calculated by Kruskal–Wallis test followed by Dunn’s post hoc test.

To examine how MC degranulation affects cochlear morphology, the supernatant from bone marrow-derived MCs (BMMCs) stimulated with mouse IgE-anti-DNP (1 µg/mL) and DNP (0.01 µg/mL) was collected. The supernatant was serially diluted (1:2000, 1:1000, 1:400, and 1:20, corresponding to 10 MCs, 20 MCs, 50 MCs, and 1000 MCs) to account for the number of MCs in the cochlear tissue [7,9] and then incubated with explants for 24 h. Immunofluorescence staining of HCs, shown in Figure 1, indicated that supernatant from activated MCs causes dose-dependent alterations in cochlear structure. We saw a progressive loss of HCs and tissue deterioration as supernatant concentrations increased. Scoring the number of hair cells showed a significant decrease in intact OHCs and IHCs after exposure to BMMC-derived supernatant, compared to control explants (Figure 2).

The scoring and analysis of the intact IHCs and OHCs in the cochlea using the Kruskal–Wallis with post hoc Dunn’s test indicated statistically significant changes. The number of intact IHCs decreased as the concentration of supernatant from degranulated BMMC increased, with significant differences between groups shown by the Kruskal–Wallis test in the apical (H(4) = 27.80, *p* < 0.0001), middle (H(4) = 30.14, *p* < 0.0001), and basal turns (H(4) = 29.35, *p* < 0.0001). Dunn’s test indicated a significant loss of IHCs at the concentrations of BMMC supernatant of 1:1000 (*p_adj_* = 0.0400), 1:400 (*p_adj_* = 0.0080), and 1:20 (*p_adj_* < 0.0001) dilutions in the apical part; 1:1000 (*p_adj_* = 0.0463), 1:400 (*p_adj_* = 0.0017), and 1:20 (*p_adj_* < 0.0001) dilutions in the middle part, and 1:1000 (*p_adj_* = 0.0319), 1:400 (*p_adj_* = 0.0029), and 1:20 (*p_adj_* < 0.0001) dilutions in the basal part, compared to the untreated control (Figure 2A–C).

Similarly, for the OHCs, a significant group effect was observed in the apical (H(4) = 31.69, *p* < 0.0001), middle (H(4) = 32.59, *p* < 0.0001), and basal parts (H(4) = 32.08, *p* < 0.0001). Dunn’s post hoc test identified a significant difference in the number of intact OHC between the control and cochlear explants exposed to the BMMC supernatant. In the apical part of the cochlea, there were significantly fewer OHCs after exposure to the BMMC supernatant diluted 1:400 (*p_adj_* = 0.005) and 1:20 (*p_adj_* < 0.0001), while the 1:2000 and 1:1000 dilutions showed no significant difference (*p* > 0.05). Similarly, in the middle turn, Dunn’s post hoc test showed a significant decrease in OHC numbers compared to the control group at the 1:1000 dilution (*p_adj_* = 0.0379), the 1:400 dilution (*p_adj_* = 0.0006), and the 1:20 dilution group (*p_adj_* < 0.0001). In the basal cochlear turn, significant OHC loss was observed in the 1:400 dilution group (*p_adj_* = 0.0005) and the 1:20 dilution group (*p_adj_* < 0.0001).

### 3.2. Compound 48/80 Stimulates the Release of Chymase and Tryptase from Cochlear Explants

**Figure 3:**
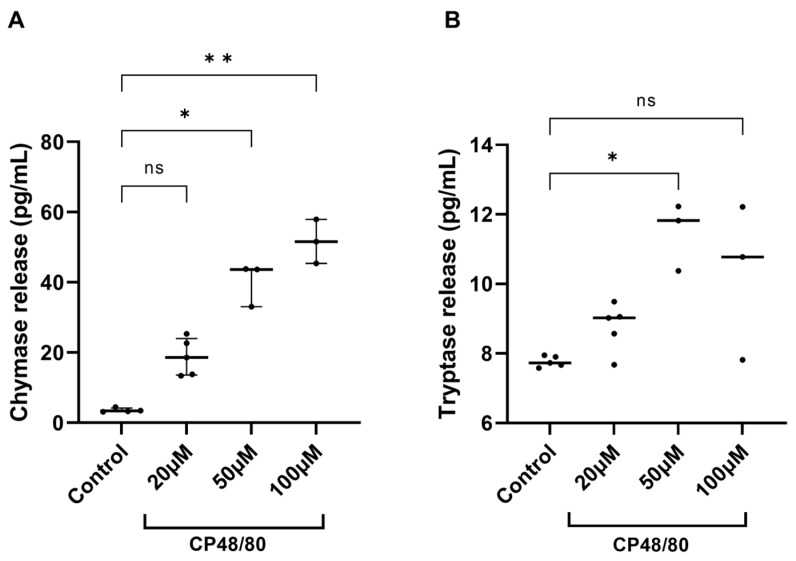
Cochlear explants release chymase (**A**) and tryptase (**B**) after exposure to CP48/80 for 30 min. The cochlear explants were exposed to 20 µM (n = 5), 50 µM (n = 3), and 100 µM (n = 3) of CP48/80 for 30 min, while the control explants were cultured in medium only (n = 4). “n” indicates the number of independent biological replicates. Data are presented as the median and the interquartile range (IQR); ns, not significant; *, *p* < 0.05; **, *p* < 0.01. The Kruskal–Wallis test, followed by Dunn’s post hoc test, was used to determine the statistical significance of the observed differences.

To assess whether resident cochlear MCs in the explants respond to stimulation with CP48/80, the explanted tissues were exposed to CP48/80 at concentrations of 20 µM, 50 µM, and 100 µM for 30 min. The control group was maintained in culture medium only. Afterwards, the supernatants were collected and assayed using ELISA. Compared to the control group, a significant release of chymase was observed in the supernatants from cochlear explants stimulated with 50 and 100 µM of CP48/80 (Figure 3A). In contrast, significant tryptase release was only detected in the 50 µM group (Figure 3B).

Because the data were not normally distributed, the Kruskal–Wallis test was used and revealed significant overall group differences in chymase (H(3) = 13.05, *p* < 0.0001) and tryptase concentration (H(3) = 9.318, *p* = 0.0088). Compared to the control group, exposure to 50 µM CP48/80 increased chymase concentration from a median 3.33 pg/mL (control) to 43.60 pg/mL (exposed explants), *p_adj_* = 0.0385. Exposure to 100 µM CP48/80 increased median chymase concentration to 51.60 pg/mL, *p_adj_* = 0.0023. A significant increase in tryptase concentration was observed only in the supernatants from cochlear explants exposed to 50 µM CP48/80 (median concentration in the controls 7.73 pg/mL vs. 11.82 pg/mL in the exposed cultures, *p_adj_* = 0.0121). CP48/80 at 20 µM and 100 µM had no effect on tryptase concentration.

### 3.3. Chymase and Histamine Release from Cochlear Explants Exposed to 15 µm of Cisplatin

**Figure 4:**
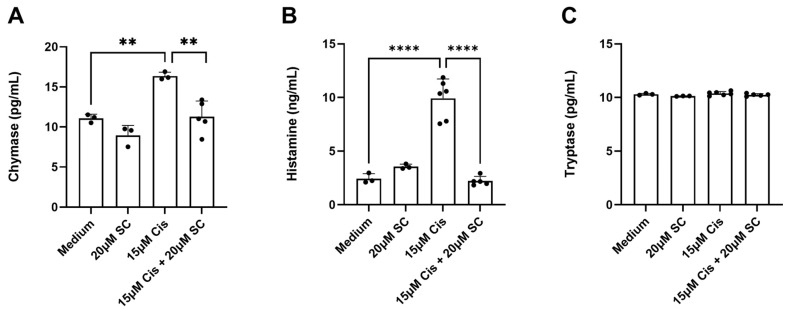
Chymase (**A**), histamine (**B**), and tryptase (**C**) release from cochlear tissue evoked by cisplatin. The control explants were either cultured in tissue culture medium for 1 h or incubated with 20 µM of sodium cromolyn (SC) for 2 h. The cochlear explants were either exposed to 15 μM cisplatin for 1 h or pretreated with 20 μM SC for 2 h, followed by exposure to 15 μM cisplatin for 1 h. The data were derived from 3 to 6 independent experiments and are presented as the mean ± SD; **, *p* < 0.01; ****, *p* < 0.0001 (one-way ANOVA followed by Tukey’s post hoc test).

To examine the ability of cochlear resident MCs to respond to cisplatin, cochlear explants were incubated with 15 µM cisplatin for 1 h at 37 °C with 5% CO_2_. Supernatants were collected to measure chymase, histamine, and tryptase, key mediators stored in MCs. The data were normally distributed and were therefore analyzed using one-way ANOVA followed by Tukey’s test. A statistically significant increase in chymase and histamine concentration was detected in the supernatant of the treated explants after 1 h of cisplatin exposure, indicating MC degranulation. Chymase release (Figure 4A): one-way ANOVA showed a significant group effect, F (3, 10) = 15.32, *p* = 0.0005, and Tukey’s post hoc test vs. control revealed a significant increase after cisplatin exposure (*p* = 0.0043), which was prevented by sodium cromolyn (*p* = 0.0026). Histamine release (Figure 4B): one-way ANOVA indicated a significant difference among groups (F (3, 13) = 51.00, *p* < 0.0001). Cisplatin induced a marked increase vs. control (*p* < 0.0001, Tukey’s test), while SC pre-treatment significantly reduced histamine release compared with cisplatin alone (*p* < 0.0001, Tukey’s test). In the same supernatants, no increase in tryptase levels was observed.

## 4. Discussion

A large portion of the Section 4 was previously published in Dr. Zeng’s dissertation [16].

MCs are essential components of the immune system, and mature MCs are found in various tissues, including the dermis [24], pulmonary alveoli and respiratory tract [18], intestinal mucosa [25], conjunctiva [26], as well as the atrial appendages of the heart [27], among others. However, it was not until 2020 that MCs in the inner ear of rodents were first identified and confirmed by the ENT research laboratory at Charité Universitätsmedizin Berlin [9]. Subsequent studies consistently demonstrated that MCs in the cochlea degranulate following cisplatin treatment, and that the administration of the MC stabilizer cromolyn provided protective effects against cisplatin-induced damage to hair cells (HCs) and spiral ganglion neurons (SGNs) [7], indicating the involvement of resident cochlear MCs in the pathological processes induced by cisplatin in the cochlea. Nonetheless, the precise mechanism remains unknown.

The primary objective of this study was to investigate the impact of MC degranulation on cochlear morphology. Subsequently, the study examined the capacity of cochlear MCs to respond to various stimuli. Finally, this investigation provided preliminary insights into the mechanisms by which MCs contribute to cisplatin-induced ototoxicity.

### 4.1. Effect of IgE/Antigen-Induced MC Degranulation on Cochlear Morphology

Epidemiological evidence has demonstrated a clinical association between allergic diseases and inner ear dysfunction [10,15,28]. To simulate an allergic environment in our in vitro experiments, we used IgE anti-DNP/DNP to induce bone marrow-derived MC (BMMC) degranulation and exposed cochlear membranous tissues to the serially diluted supernatants from these experiments. Our findings indicate that even at minimal concentrations, there exists a distinct, concentration-dependent trend of HC loss and damage to tissue architecture. As the concentration of the supernatant increased, the initial orderly arrangement of HCs became progressively disrupted, and the number of intact cells diminished substantially. It is also important to note that the sample sizes in these experimental groups may have limited the statistical power to detect more subtle effects at lower concentrations, even after strict correction for multiple comparisons. Nonetheless, the consistent and highly significant damage observed at higher concentrations (1:400 and 1:20 dilutions) offers strong evidence for the ototoxic potential of MC mediators.

Our findings emphasize the possible link between allergic diseases, MC activation, and hearing loss, although the causative nature of this connection and its clinical relevance remain to be proven. The experiments, in which guinea pigs were passively sensitized and then locally challenged with the antigen injected into the stylomastoid foramen, have demonstrated increased threshold and wave I peak latency in the auditory brainstem responses, nystagmus, and a negative summating potential on electrocochleography [29,30,31]. Importantly, all these symptoms could be alleviated by tranilast, an inhibitor of MC degranulation [30]. In addition, using pranlukast hydrate or a histamine H1-receptor antagonist administered before antigen challenge could also prevent the inner ear reaction, suggesting that MC mediators, such as leukotrienes and histamine, may play a key role in the process [32,33]. Other MC mediators, such as cytokines and proteases (including chymase and tryptase), can contribute to inflammation and tissue damage [34,35]; thus, inducing long-term damage. In the inner ear, where delicate structures like HCs are crucial for auditory function, any disruption in morphology can have significant consequences for hearing.

### 4.2. Effect of Compound 48/80 Stimulation on Cochlear MCs

CP48/80 is a classic MC activator that induces MC degranulation by interacting with the Mrgprb2 receptor on MC membranes [36]. Mrgprb2, the mouse equivalent of the human MRGPRX2, and MRGPRX2 are receptors mainly studied in relation to non-IgE-mediated MC degranulation and inflammation [36,37,38]. The activation of Mrgprb2 and MRGPRX2 by neuropeptides, such as Substance P, and other polybasic secretagogues, including CP48/80, results in IgE-independent anaphylactic reactions [36,39,40]. Thus, we used CP48/80 in our second research objective to evaluate the mediators released by resident cochlear MCs in response to CP48/80 stimulation. The results depicted in Figure 3 revealed significant chymase release at 50 µM and 100 µM, while tryptase release reached statistical significance only at 50 µM. This differential release pattern could be attributed to distinct granule populations or to differences in release kinetics. Additionally, the small sample sizes, especially in the 50 µM and 100 µM groups (n = 3), probably decreased the statistical power to identify smaller effects. Limited incubation time could also be considered as a reason. Future studies with larger sample sizes and longer culture times would be valuable for confirming these patterns and more precisely defining the release kinetics of different MC proteases in the cochlea.

### 4.3. The Effect of Cisplatin on Cochlear MCs

The final part of our study shows that cisplatin exposure quickly activates cochlear resident MCs, indicated by the significant release of chymase and histamine into the extracellular space. This emphasizes the potential role of MCs as early responders to ototoxic insults and highlights their part in the pathophysiological process initiated by cisplatin in the cochlea.

The concentration of cisplatin (15 µM) used in our in vitro experiments is consistent with the findings from the animal model. Laurell et al.’s work has demonstrated that intravenous administration of cisplatin at 12.5 mg/kg in adult guinea pigs results in a perilymph concentration of 4.2 µg/mL (14 µM) 20 min after injection, which aligns with the concentration used in our studies [41]. Additionally, recent research by Breglio et al. demonstrated the long-term retention (months to years) of cisplatin in the cochleae of mice and humans [42]. However, the cisplatin concentration was not measured in the cochlear fluids (perilymph, endolymph) during these experiments; instead, it was measured in the tissues (stria vascularis, organ of Corti, spiral ganglion). The translational relevance of these findings still needs to be confirmed clinically; however, designing such a trial is challenging due to ethical concerns. So far, the only supporting experiments come from studies using human postmortem specimens [42].

The observed increase in chymase and histamine in the supernatant of cisplatin-treated cochlear explants aligns with the canonical activation profile of MCs, which undergo degranulation to release preformed mediators upon stimulation [34,35].

Chymase, a serine protease, is a well-established marker of MC activation and has been implicated in the mechanisms of inflammation and allergy, angiogenesis, oncogenesis, remodeling of the extracellular matrix in connective tissue, and changes in organ histoarchitecture [43]. Additionally, the rapid release of MC proteases in innate immune responses is associated with enhanced host defense and/or increased survival, for example, by increasing resistance to parasites and degrading endogenous or exogenous toxic peptides [44,45,46,47]. Experimental evidence from two independent investigations using murine models of nematode infection has revealed a significant expansion of MC populations within the intestinal mucosa during parasitic challenge. These studies further demonstrate that infection-induced MC activation triggers the secretion of chymase-containing mouse MC protease-1 (mMCP-1) [47,48]. The release of mMCP-1 can contribute to nematode expulsion by disrupting the epithelial barrier through the degradation of the tight junction protein occludin [47,48]. Histamine, a biogenic amine, exerts pleiotropic effects on vascular permeability [34,35] and neuronal signaling [49], which may exacerbate cochlear damage in cisplatin-induced ototoxicity. The attenuation of these mediators in explants pretreated with the MC stabilizer sodium cromolyn (SC) further corroborates the specificity of cisplatin-induced MC activation. This pharmacological rescue suggests that cromolyn may serve as a protective agent against early MC-mediated cochlear inflammatory responses, warranting further investigation into its therapeutic potential.

Notably, the absence of detectable tryptase in the supernatants introduces an intriguing dimension to our findings. Tryptase, a serine protease stored in MC granules alongside chymase, is often regarded as a biomarker for systemic MC activation [34,35]. Its absence in this context may reflect tissue-specific heterogeneity in mediator release by MCs. Cochlear MCs, those residing in the modiolus and the spiral ligament [7,9], may exhibit a distinct granule composition compared to MCs in other tissues, favoring chymase over tryptase. Alternatively, the kinetics of tryptase release may differ under cisplatin stimulation, with delayed secretion occurring beyond the 1 h experimental window. This discrepancy underscores the need for temporal studies to map the dynamic release profiles of MC mediators in response to ototoxic agents.

The rapidity of cisplatin-induced MC degranulation (within 1 h) challenges the conventional view of cisplatin ototoxicity as a delayed process primarily driven by oxidative stress and apoptosis [50,51,52]. Our data suggest that MC activation may represent an upstream event that primes the cochlear microenvironment for subsequent damage. For instance, histamine could potentiate inflammatory cascades via H1/H4 receptor signaling on resident macrophages or spiral ganglion neurons, while chymase might directly damage sensory HCs by degrading intercellular junction proteins. Furthermore, MC-derived mediators could disrupt the blood-labyrinth barrier, facilitating the entry of pro-apoptotic cisplatin metabolites into the cochlear compartments. Further research is needed to clarify how cisplatin influences MC function and its role in cisplatin-related ototoxicity and hearing loss. Uncovering these mechanisms could lead to new strategies to reduce cisplatin’s harmful effects on hearing and enhance how chemotherapy-related hearing loss is managed.

The therapeutic implications of targeting MCs in cisplatin ototoxicity are twofold. First, the efficacy of SC in suppressing chymase and histamine release supports the rationale for repurposing MC stabilizers as adjunctive therapies during cisplatin chemotherapy. Second, the lack of tryptase elevation suggests that cochlear MCs may represent a unique pharmacological target, minimizing off-target systemic effects associated with broad-spectrum MC inhibitors. However, the translational relevance of these findings requires validation in vivo, where systemic factors such as plasma extravasation and immune cell infiltration may modulate MC activity. Additionally, since cisplatin causes systemic electrolyte imbalance [53] due to kidney damage, which could be prevented in MC-deficient mice or minimized in wild-type animals using cromolyn sodium [54], it is expected that systemic electrolyte disturbances could also be reduced either by the absence of MCs or by cromolyn sodium. However, the local direct damage to the stria vascularis caused by cisplatin, resulting in a lowered potassium concentration in endolymph [55], is unlikely to be mitigated by cromolyn sodium. Still, a possible reduction in the extent of systemic electrolyte imbalance, which contributes to cisplatin-induced hearing loss, should be addressed in future studies with cromolyn sodium.

Our study establishes MC activation as an early and targetable event in cisplatin-induced cochlear injury. The chymase-histamine axis emerges as a critical mediator of this process, offering novel biomarkers and therapeutic avenues for mitigating ototoxicity. These findings underscore the importance of neuroimmune mechanisms in hearing loss and support the need for further investigation of MC-centric strategies to preserve auditory function.

### 4.4. Limitations and Future Directions

The limitations of our study primarily stem from the research methodology. First, in the initial phase, when the supernatant from degranulated BMMC was used to induce cochlear morphological changes, identifying the specific mediators responsible for the damage was difficult. During MC degranulation, various mediators, including histamine, serotonin, chymase, tryptase, and multiple cytokines, are released, resulting in a complex sequence of effects throughout the 24 h culture period. Future research employing specific inhibitors or isolated mediators could help clarify the signaling pathways involved in MC-associated cochlear damage, offering a deeper understanding. Second, the absence of a reliable way to isolate MCs from the inner ear was a major limitation. As a result, all experimental findings provide only indirect evidence of the role of cochlear MCs in both normal and disease states. Subsequent research should focus on methods enabling functional in situ single-cell analyses. Furthermore, the ex vivo explant model, although valuable for examining short-term cochlea-specific responses, lacks the systemic immune and vascular interactions that occur in vivo. Additionally, the 15 µM cisplatin concentration, although consistent with previous in vitro ototoxicity studies, may not accurately reflect the cochlear pharmacokinetic profile of cisplatin. Future research could use single-cell transcriptomics to identify MC subpopulations in the cochlea and their varied responses to cisplatin. Moreover, our research did not explore the role of ROS as a second messenger in MC activation or examine how inhibiting ROS might affect cochlear MC degranulation, which should be investigated in future studies. Finally, to determine the role of membrane-bound molecules (e.g., adhesion molecules) in activated BMMCs on cochlear tissues, future experiments should use a coculture system.

Despite advances in research on inner ear immunology, critical knowledge gaps persist. First, the precise mechanisms by which MC activation initiates or exacerbates cochlear damage remain undefined. While cisplatin rapidly induces MC degranulation in vitro, it is unclear whether this represents a primary insult or a secondary response to oxidative stress [56]. Second, the heterogeneity of MC mediator release in the cochlea—evidenced by the tissue-specific absence of tryptase after cisplatin exposure—suggests compartmentalized immune regulation; however, the molecular drivers of this selectivity remain unexplored. Third, although MC stabilizers like SC mitigate cisplatin-induced HC loss, their therapeutic potential in combination regimens remains untested.

## 5. Conclusions

This study demonstrates that cochlear morphology in murine models, particularly the integrity of HCs, is compromised by degranulated MCs, suggesting a mechanistic connection between MC-mediated pathologies and inner ear dysfunction. Clinical correlations may emerge from epidemiological analyses of hearing loss prevalence in populations with allergies. Notably, cochlear-resident MCs exhibited marked sensitivity to Compound 48/80 stimulation despite having a low baseline abundance. Activation of these MCs triggered chymase release during cisplatin exposure, which could be inhibited by the MC stabilizer sodium cromolyn (SC). These findings align with prior work from the Charité Otolaryngology Research Laboratory, which demonstrates cromolyn’s protection against cisplatin-induced HC loss [7], suggesting that MCs may initiate ototoxic pathways before the accumulation of reactive oxygen species.

This work advances our understanding of cochlear MC biology and its dual role in inner ear immunity and pathology. While MCs are established mediators of allergic inflammation, their specific contributions to auditory damage mechanisms warrant systematic exploration. Future studies should prioritize characterizing MC-derived mediators and biochemical cascades to develop targeted therapies for cisplatin-induced hearing loss.

## Data Availability

The original contributions presented in this study are included in the article. Further inquiries can be directed to the corresponding author.

## Abbreviations

The following abbreviations are used in this manuscript:

ALHL	Acute low-tone hearing loss
BMMC	Bone marrow-derived mast cell
CIHL	Cisplatin-induced hearing loss
CP48/80	Compound 48/80
DAPI	4′,6-Diamidino-2-phenylindole
DMEM	Dulbecco’s Modified Eagle Medium
DMSO	Dimethyl sulfoxide
DNP	Dinitrophenol
ELISA	Enzyme-linked immunosorbent assay
HC	Hair cell
IHC	Inner hair cell
ISSHL	Idiopathic sudden sensorineural hearing loss
MC	Mast cell
MD	Ménière’s disease
mMCP-1	Mouse mast cell protease-1
MRGPRX2, Mrgprb2	Mas-related G protein-coupled receptor X2
OC	Organ of Corti
OHC	Outer hair cell
RT	Room temperature
SC	Sodium cromolyn
SEM	Standard error of the mean
SNHL	Sensorineural hearing loss
